# Volatile Compounds in Green and Roasted Arabica Specialty Coffee: Discrimination of Origins, Post-Harvesting Processes, and Roasting Level

**DOI:** 10.3390/foods12030489

**Published:** 2023-01-20

**Authors:** Fosca Vezzulli, Milena Lambri, Terenzio Bertuzzi

**Affiliations:** 1 Department for Sustainable Food Process, DiSTAS, Università Cattolica del Sacro Cuore, Via Emilia Parmense 84, 29122 Piacenza, Italy; 2 Department of Animal, Nutrition and Food Sciences, DIANA, Università Cattolica del Sacro Cuore, Via Emilia Parmense 84, 29122 Piacenza, Italy

**Keywords:** coffee aroma, volatile compounds, headspace analysis, roasting, coffee origin, specialty coffee

## Abstract

The aroma of coffee is a complex mixture of more than 1000 compounds. The volatile compounds in green and roasted coffee were analyzed to detect several features related to quality, roasting level, origins, and the presence of specific defects. With respect to specialty coffee, the flavor profile and peculiarities of the aforementioned characteristics are even more relevant knowing the expectations of consumers to find, in a cup of coffee, unicity bestowed by its origin and post-harvesting processes. In this work, which dealt with 46 lots of specialty Arabica coffee, we used HS-SPME/GC–MS to detect the volatile compounds in green coffees together with those in the same coffees roasted at three different levels to identify whether differences in headspace composition were ascribable to the origin, the post-harvesting processes, and the roasting profiles. The main results are related to the discriminant power of the volatile compounds in green coffee, which are impacted by the origins more than the post-harvesting processes. Compounds such as linalool and 2,3-butanediol were more concentrated in natural coffees, while hexanal was more concentrated in washed varieties (*p* < 0.05). In roasted coffees, the differences in composition were due to roasting levels, countries of origin, and the post-harvesting processes, in descending order of significance.

## 1. Introduction

As with many of the best-loved foodstuffs, coffee and coffee beverages are chosen and identified first by their unique aroma, which plays a crucial role as a marker of coffee quality and characteristics, both for green and roasted coffee [1]. A number of chemical compounds, especially volatile compounds, are involved in the flavor profile of roasted coffee. More than 1000 volatile compounds (mainly produced by Maillard reactions) of several chemical classes have been identified in roasted coffee, but only a small percentage (about 5%) play a relevant role in coffee aroma [2]. Consumers are increasingly attracted to single-origin coffees rather than blends thanks to the specific flavor profile these coffees can offer [3]. The exponential increase in demand for one such highly traceable coffee, known as Arabica Specialty coffee, with its certified origins, varieties, and post-harvesting processes [4], has played a crucial role in the investigation into the impacts these parameters have on the volatile profile of both green and roasted beans [5].

The composition of the volatile fraction in coffee is influenced by many factors, such as the variety, the agro-ecological zone of cultivation (climate, soil, altitude, etc.), the post-harvesting processes (e.g., fermentation, washing, and drying), and the roasting and brewing parameters (temperature, pressure, etc.). The characterization of the volatile fraction has helped to identify defects [6,7,8], the different roasting levels [9], the origins of [10,11] raw or roasted coffee, and the influence of altitude and the climatic conditions of farming [12,13] on raw and roasted coffee. Some studies have also evaluated the influence of the post-harvesting processes on the volatile compounds found in coffee [14,15].

Headspace solid-phase micro-extraction (HS-SPME) coupled with gas chromatography–mass spectrometry (GC–MS) is often used in volatile fraction characterization [16,17]. Moreover, composition data can be processed to evaluate the possible relationships between volatile composition and specific factors of coffee, such as origin, post-harvesting processes, and roasting levels [18].

In this context, we carried out HS-SPME/GC–MS analysis of 46 specialty Arabica coffees from Asia, Africa, Central, and South America, including green samples and samples roasted at three different levels. Volatile composition results were then processed to evaluate whether they were relevant in the discrimination of coffee origin, the post-harvesting processes used to remove the pericarp of the fruit from the beans (natural, honey, and washed methods, also known as dry, semi-dry, and wet methods), and the roasting levels. Finally, the correlations among volatile compounds occurring in green and roasted coffee were studied.

## 2. Materials and Methods

### 2.1. Sampling

In all, 46 samples of Arabica green coffee of the 2020–2021 crop, originating from Brazil, Burundi, Colombia, Costa Rica, Democratic Republic of Congo, Dominican Republic, El Salvador, Ethiopia, Guatemala, Haiti, Honduras, India, Indonesia, Kenya, Peru, Republic of Panama, Rwanda, and Uganda were shipped in 60 kg GrainPro bags, sampled by a local supplier, and delivered to our laboratories. All Arabica coffees were graded as “Specialty” or “Premium” coffees according to the protocol established by the Specialty Coffee Association. Specifically, the samples were required to have a cupping score of 80 points or more and needed to be free of primary defects (sour beans, foreign matter, or insect/fungus damage). Only a small number of unripe or broken seeds were allowed. Coffee samples represented 30 of the different varieties of Arabica cultivated in the countries of origin, increasing the variability and representativeness of the sample sets. In the post-harvest processes, 17 samples were subjected to the dry process, 4 to the semi-dry process, and 25 to the wet process [19].

A total of 500 g of green coffee was frozen, milled using a cyclone hammer mill (1 mm sieve, Pulverisette, Fritsch GmbH, Idar-Oberstein, Germany), and homogenized. Then, an aliquot of 2 g was stored at −20 °C until the time of analysis.

After 3 different roasting processes (see Section 2.2) of each Arabica green coffee sample, 3 different samples of roasted coffee were obtained for a total of 138 samples of roasted coffee.

### 2.2. Roasting

The sample roaster IKAWA Model V2-PRO was used to roast all the coffee samples as described by Vezzulli et al. [16]. Each roasting batch was 50 g (±0.5 g). Three roasting profiles, referred to in this work as light, medium, and dark, reached roasting levels comparable to the industrial levels between the first and second crack [19]. The chamber was preheated to 174–175 °C before the coffee was dropped in. Light roasting was achieved at 205 °C in 5.46 min, medium roasting at 210 °C in 6.16 min, and dark roasting at 214 °C in 6.46 min. After being discharged from the roasting chamber, samples were cooled and then ground using a Moulinex blender (Model AR110830). The heating of the beans was minimized during the milling. Immediately after this, 2 g of ground coffee was placed in a 15 mL vial, closed by a crimp cap with a Teflon-lined septum (Supelco, Bellefonte, PA, USA), and frozen until analysis.

### 2.3. Analysis of Volatile Compounds

The volatile compounds in the coffee samples were detected using an HS-SPME/GC–MS system (Thermo-Fisher Scientific, San Jose, CA, USA) according to previous studies [11,18,20]. After being defrosted at 5 °C and stabilized at 20 °C, each sample was incubated at 50 °C under agitation for 10 min. Then, the headspace was sampled (30 min) using an SPME fiber coated with DVB/CAR/DMS (75 µm) (Supelco, Bellefonte, PA, USA), pre-conditioned according to manufacturer recommendations, at 50 °C for 30 min under continuous agitation. Afterward, the fiber was thermally desorbed at 220 °C for 3 min in splitless mode. The volatile compounds were analyzed using a TraceGQ Ultra coupled with an ISQ single quadrupole mass spectrometry (Thermo-Fisher Scientific, San Jose, CA, USA). The volatile compounds were separated on a capillary column Rtx-5MS, 30 m × 0.25 mm i.d., 0.25 µm film thickness (Restek Corporation, Bellefonte, PA, USA). Helium, the carrier gas, was maintained at a constant flow rate of 1.0 mL/min. The oven temperature was set at 40 °C for 3 min. Then, the temperature was increased to 180 °C at 12 °C/min and held for 5 min. Finally, the temperature was increased to 240 °C at the rate of 40 °C/min up and held for 5 mins. The transfer line temperature was set at 250 °C and the MS source at 250 °C. Mass spectra were acquired in the electron impact mode at 70 eV, using an *m*/*z* range of 50–650. When reference compounds were not available, compounds were identified after comparing the mass spectra with the NIST database. The linear retention indices (LRIs) of the volatiles were compared with data from the literature. ThermoQuest Xcalibur 1.2 software was used to obtain the data. The results were expressed as the relative percentage of each compound’s peak area to the sum of the identified GC–MS peak area. Each analysis was carried out in duplicate. The absence of contaminants was verified by the injection of a blank sample every three injections.

### 2.4. Statistical Analysis

Statistical analysis of the volatile compounds was conducted using the IBM SPSS statistics software (ver. 27, Inc., Chicago, IL, USA). The homogeneity of variance was checked. A *t*-test was applied to discriminate between the set of data from Ethiopian and American coffees and between washed and natural samples. One-way ANOVA with a Waller–Duncan post-hoc test and discriminant analysis was applied to evaluate the significant differences among coffees belonging to the three Arabica subgroups (natural, honey, and wet), among coffees from different continents and countries of origin, and among coffees roasted at different levels. Data from the volatile composition obtained via the headspace analysis of the green and roasted coffee samples, expressed in terms of area under the chromatogram, were statistically treated via discriminant canonic analysis to detect whether the volatile profile allowed one to discriminate among coffees from different countries of origin, coffees that had undergone different post-harvesting processes, and coffees roasted to different levels.

## 3. Results

### 3.1. Volatile Compounds in Green Coffee

In our study, HS-SPME/GC–MS analysis helped detect 51 compounds, which were divided into 12 chemical groups (Table 1). The hydrocarbon group was the most numerous (n = 13). However, this group was difficult to identify due to the similar mass spectrum of some isomers and the lack of standards. For this reason, only the number of carbon atoms was reported. The composition of the volatile fraction showed different profiles among the samples. In each coffee sample, 23 to 40 different compounds were detected; 8 compounds were detected in not more than 5 samples, whereas 35 were detected in more than 20 samples. No relevant differences were found between the average number of compounds detected in dry and wet coffees (35 vs. 34).

Considering the markers of defective beans, benzaldehyde was detected mainly in dry coffee (12 of 13 samples), whereas 2-methylpyrazine was detected in 14 samples (5 dry and 9 wet coffees).

### 3.2. Volatile Compounds in Roasted Coffee

After samples were roasted at three different levels, the volatile compositions of the obtained samples were again analyzed via headspace analysis, and the values were expressed in terms of area under the chromatogram.

Origin and roasting conditions significantly influence flavor formation and aroma quality. It is well known that roasting can modify, modulate, and generate the final aroma of coffee, generating more than 100 compounds [20,21,22,23,24,25,26,27].

A total of 70 compounds were identified and, after validation, 56 were included in the analysis, where they were divided into nine chemical groups (Table 2). Furan was the most numerous group (n = 19). Almost all the compounds were detected in all the samples. However, the abundance of 10 compounds was discriminant for each of the three roasting levels (pyridine; furfural; furfuryl alcohol acetate; 2,4-dimethyl-1,3-cyclopentanedione; 1-methyl-2-acetonirtrile-pyrrole; maltol; 1,2,-furanyl-methyl-1-pyrrole; furan-5-methyl-2,2-methylendi; furfuryl-3-methyl-butanoato; and 2,2-oxydimethylen-difurane), their concentration increasing with the roasting level, as already reported by Moon et al. [28].

Only one compound (3,5-dietil-2-metil pyrazine; floral odor) decreased with an increasing roasting level.

## 4. Discussion

### 4.1. Green Coffee Origin and Discrimination and Characterization Based on the Post-Harvesting Processes

Volatile compounds in green coffee have been widely studied for detecting defective elements and the influence of altitude or climatic conditions. Specifically, the presence of defective beans, such as black, sour, immature, or moldy beans deriving from inappropriate agricultural, harvesting, and post-harvesting practices, impacts the volatile compounds profile of a coffee lot. Cantergiani et al. [6] identified some compounds causing a moldy/earthy flavor in coffee and suggested their presence as being influenced by post-harvest drying. Toci et al. [7] identified 2-methylpyrazine and 2-furylmethanol acetate in black immature beans as well as benzaldehyde and 2,3,5,6-tetramethylpyrazine as markers of defective beans in general. Bertrand et al. [13] reported that butan-1,3-diol and butan-2,3-diol are correlated with acidity, a reduction in aroma quality, and an increase in earthy and green flavors. Moreover, they assumed that high temperatures induce higher levels of these compounds.

**Figure 1:**
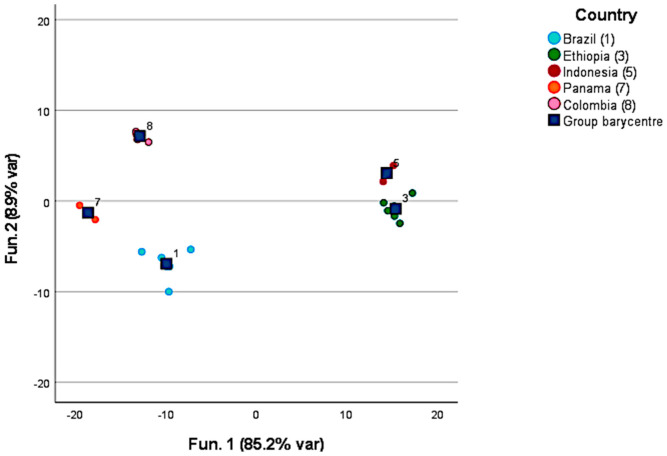
Discrimination of green coffee samples by the countries of origin.

Figure 1 provides the results of the discrimination of green coffee samples by country of origin. Only countries represented by more than five samples in the sample set were considered (n = 29). The discriminant compounds included in the model are ethanol; 1-methoxy-2-propanone; isovaleric acid methyl ester; hexanal; 2,3-butanediol; methylpyrazine; butanoic acid 2-methylethyl ester; isovaleric acid ethyl ester; xylene; isovaleric acid; butanoic acid 2-methyl; pinene; alpha-pinene; methyl nonane; benzaldehyde; pentanoic acid 3-methyl; 2-pentylfuran; n-decan; octanol; carene; and 2,6-dimethyloctane. The two-function model was able to correctly group 100% of the samples. However, unfortunately only a small number (<15%) of the samples were correctly identified by the leave-one-out validation. This can be justified by the fact that the aroma profile of a coffee increases in complexity and intensity once it is roasted since the precursors, and some fixed compounds, undergo Maillard and browning reactions originating new volatile molecules.

A *t*-test was conducted to identify differences in the composition of the volatile fractions in American and African coffee samples. In this respect, American samples were richer in methyl ester isovaleric acid, tetramethyl octane, and 3-methoxypyrazine-2-isobutyl, while the African samples were more concentrated in 2-methyl butanoic acid, pinene, and D-limonene (*p* < 0.05).

Additionally, an ANOVA combined with a Waller–Duncan post hoc test showed that Asian coffees were the richest in 3-methylbutanol, 2-pentylfurane, and 3-hydroxybutanoic acid 2,2,4-trimethylpentil ester. Conversely, African coffees were characterized by pinene and D-limonene and the American coffees showed to be the least concentrated in butanoic acid.

**Figure 2:**
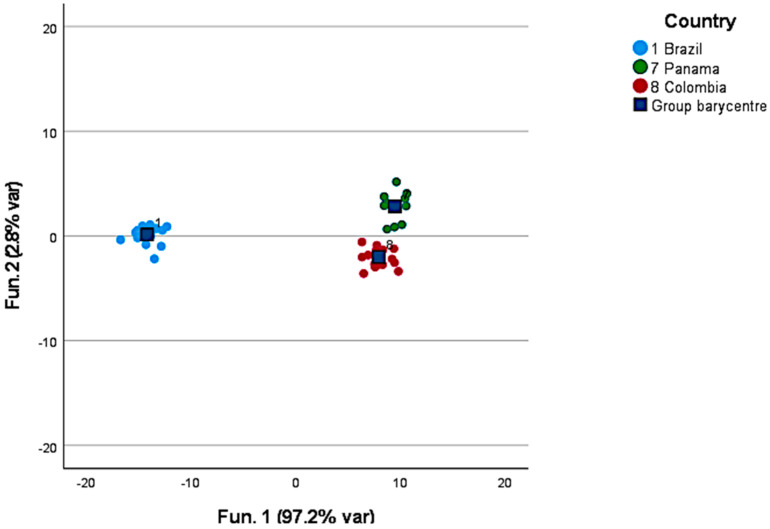
Discrimination of Brazilian, Colombian, and Panamanian green coffee in the American subcluster.

To improve the reliability of the discrimination, the variables in the model were reduced by selecting samples only from one continent at a time. Figure 2 provides the results obtained when discriminating samples from three American countries: Brazil, Colombia, and Panama. The discriminant compounds were, in this case, ethanol, methyl nonane, n-decan, D-limonene, and tributyl phosphate. All the samples (100%) were properly clustered, and contrary to the earlier result, 95.8% of the samples were also correctly associated after leave-one-out validation. Guyot et al. [29] found that coffee from higher elevations in Guatemala exhibit higher beverage quality, successively confirmed in several Central American countries [12,30,31]. Tsegay et al. [32] identified volatile compounds in Ethiopian coffee samples and found only weak correlations with the altitude of the cultivation area.

Interestingly, reducing the variability to only a post-harvesting process, the continent of origin was also discriminated considering only washed processed samples. The model, built on the concentrations of ethanol; butanoic acid 2-methyl; 2,6-dimethyloctane; D-limonene; phenylethyl alcohol; 3-methoxypyrazine-2-isobutyl; and 3-hydroxybutanoic acid 2,2,4-trimethylpentil ester, was able to properly cluster 88.6% of the samples and 77.1% of them were also validated via the leave-one-out test.

**Figure 3:**
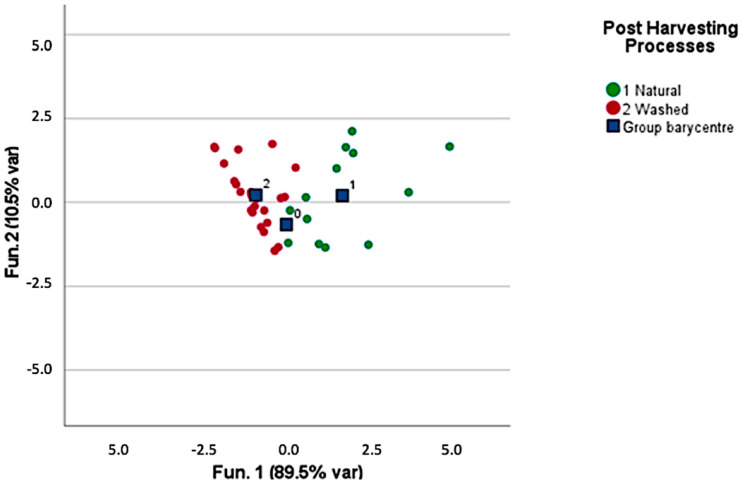
Discrimination between washed and natural post-harvesting processed green coffee samples.

Dealing with the post-harvesting processes, discriminant canonic analysis was performed to classify the samples obtained via natural and washed processes. Honey, pulped, and anaerobic processes were not included in the model due to their variability and the small number of samples available. Figure 3 provides the clustering obtained by modeling the concentration of 2,3-butanediol; pinene; and octanol, which turned out to be the discriminant molecules for the post-harvesting processes. In the model, 77.8% of the samples were correctly clustered and the leave-one-out validation also provided the same discriminant power.

As per Gonzalez-Rios et al. [14], the volatile fraction of green coffee beans is primarily given by the alcohols, acids, esters, and aldehydes which are mainly formed during the fermentation stage of the post-harvesting process. Acids and aldehydes may also be formed during drying. They also reported that the fermentation stage increases the volatile compound fraction, particularly if fermentation was carried out in water.

*T*-test was also performed to compare washed and naturally processed coffees. Washed coffees appeared to have lower concentrations of acetic acid; 2,3-butanediol; 3-methylpentanoic acid; octanol; furfuryl alcohol; alpha-linalool; and 3-methoxypyrazine-2-isobutyl. However, they had higher amounts of hexanal, 3-methylundecane, and undecane. The results were the same for both the entire set of samples and the test within a single-origin subset. The outcomes can be justified considering the solubility of the different chemical classes that can alternatively be lost or not during the washing process, which consists of a long fermentation period (24 to 48 h) for the coffee beans in an aqueous medium. As per Bertrand et al. [20], 2,3-butanediol and hexanal showed a high correlation of the earthy attributes of acidity and bitterness, respectively. The fact is supported by a study by Marin et al. [33] which reported that in coffees, hexanal can be produced by the oxidative degradation of unsaturated fatty acids during storage, and in wet coffees, it can be formed during the long stage of washing.

Finally, results showed that the wet process probably reduced the compounds belonging to the butanoic class (methyl butanoate, 3-methyl butanoic acid, 2-methyl butanoic acid, and 3-methyl-but-2-en-oic acid). Considering samples from the same origin, such as Ethiopian coffees, the reduction in butanoic compounds for wet samples was more than 50% (−57.2%). Our results confirmed the recent findings of Elhalis et al. [15], which evaluated the effect of wet fermentation on flavor volatiles. In our study, we found a significantly higher level of hexanal and a lower level of butanoic acid in wet green coffee.

### 4.2. Discrimination and Characterization of the Origin, Post-Harvesting Processes, and Roasting Level of Roasted Coffee

Data were statistically treated via discriminant canonic analysis to detect whether the volatile profile allowed one to discriminate among different countries and continents of origin, roasting levels, and post-harvesting processes.

**Figure 4:**
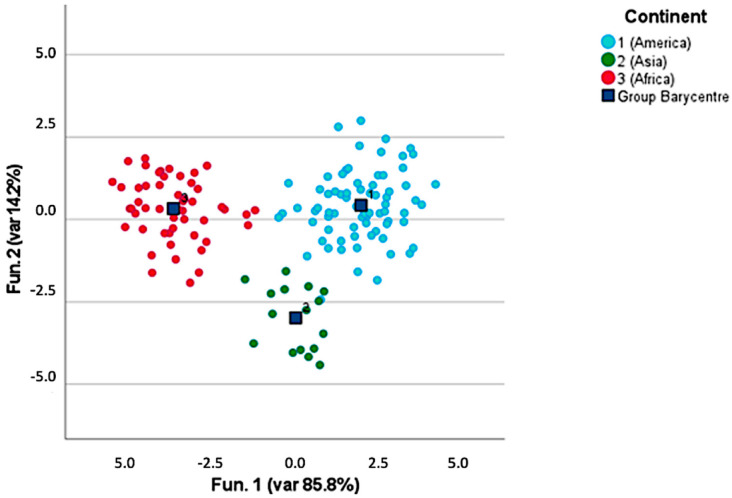
Discrimination of the continents of origin of roasted coffee samples.

Figure 4 provides a general overview of the distribution of the coffee samples after three different roasting processes in clusters homogeneous for the continent of origin of the green coffee. It can be stated that, independently of the roasting level, coffee kept a certain degree of unicity deriving from its area of origin. The discriminant functions were built on the concentrations of discriminant volatile molecules, such as pyridine; methyl pyrazine; furfuryl alcohol; isovaleric acid; furfuryl formate; furfuryl alcohol acetate; 2-methyl-6-ethyl pyrazine; benzene acetaldehyde; 2,4-dimethyl-1,3-cyclopentanedione; 3-ethyl-2,5-dimethyl pyrazine; furan-2,2-methylenbis; 3-furanone-4-hydroxy-2,5-dimethyl; 3-methyl-2,4-pentanedione; pyrazole-3,4-pyrimidine; 2-acetyl-3,5-dimethyl pyrazine; 3-phenylfurane; 5,6-methylfuran-2-yl-hexan-2-one; and ethyl-pentamethyl-benzene. Discriminant canonic analysis was able to correctly classify 98.6% of the samples, and 95.8% of the samples were also validated via the leave-one-out test. Our results are in line with those already provided by different authors about pyridine as a discriminant molecule for identifying the continent of origin of coffee, together with its impact on the coffee sensory profile delivering roasted and nutty aromas [10,11,20,34].

The discrimination of the roasting levels of none of the samples was satisfactory in terms of the classification of samples (<90% of correct association). Therefore, discrimination of subclusters homogeneous for the continent of origin and the post-harvesting process was conducted.

**Figure 5:**
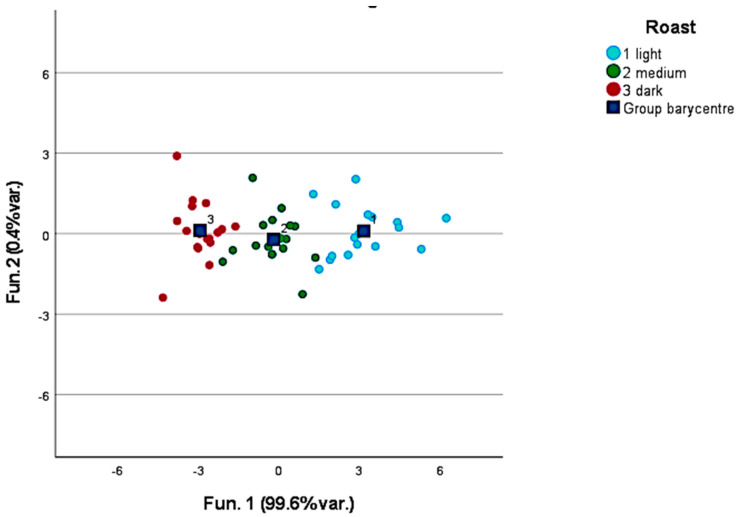
Discrimination of roasting levels in the African coffee subcluster.

**Figure 6:**
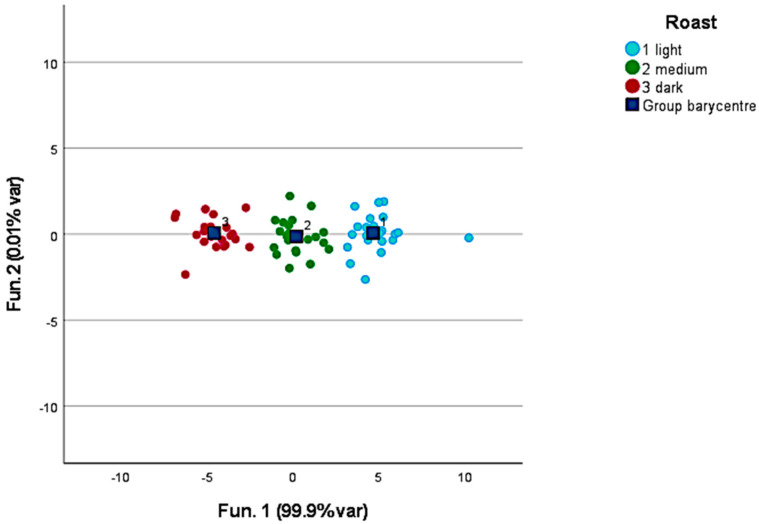
Discrimination of roasting levels in the washed coffee subcluster.

Figure 5 and Figure 6 provide the graphical results of the canonic discriminant analyses conducted on African coffees (Figure 5), and washed samples (Figure 6).

The former discrimination showed that furfuryl alcohol; 5-methyl-2-furfuraldehyde; furfuryl alcohol acetate; and 3,5-diethyl-2-methyl pyrazine were the discriminant molecules for the roasting level among samples of the same origin. The latter identified 1-furfuryl-etanone; 2,4-dinethyl-1,3-cyclopentanedione; 2,2-methylenbisfurane; 3-furanone-4-hydroxy-2,5-dimethyl; 3-methyl-2,4-pentanedione; 3,5-diethyl-2-methyl pyrazine; 1,2-furanylmethyl-1-pirrol; and damascenone. Both models correctly clustered 90% of the samples and were also confirmed by the leave-one-out validation.

It was difficult to identify post-harvesting processes on the basis of the volatile profile of coffee. To enhance the discriminant power, the analysis was conducted considering only one roasting profile at a time, and the best discrimination was obtained on light-roasted coffee, explained by the fact that the lighter the roast, the more preserved the acidic and volatile profile deriving from the fermentation/drying phases. Irrespective of any improvements, only 79.2% of the samples were classified in the proper post-harvesting process group, and 68.8% of them were validated. These poor results must also be considered in light of the small number of discriminant molecules, such as methylpyrazine; 3,5-diethyl-2-methylpirazyne; and 2,2-oxydimethylen-difurane.

**Figure 7:**
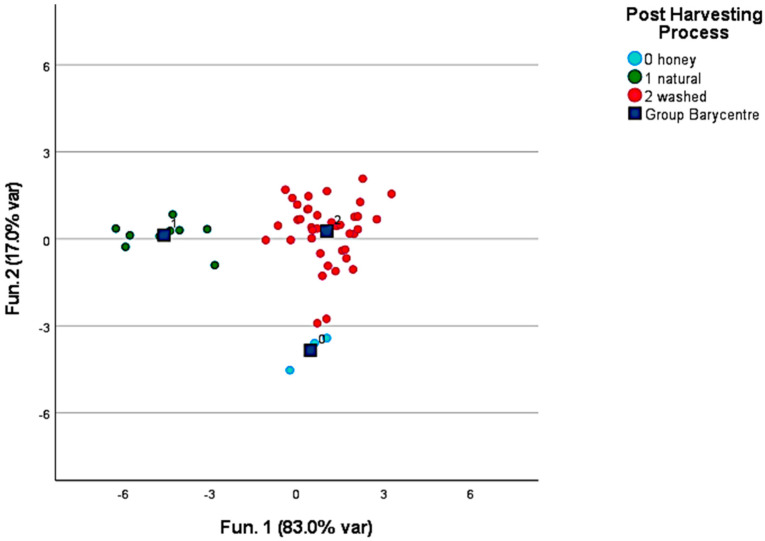
Discrimination of post-harvesting processes in the African coffee subcluster.

As the same issues in post-harvesting process discrimination were outlined by Caporaso et al. [18], and tentatively connected with variability provided by origins, a discriminant model was built considering only African (Figure 7) coffees at different roasting levels, and 96.1% of the samples were correctly classified with 96.1% confirmed after cross-validation.

A *t*-test (*p* < 0.05) was conducted to identify any significant differences in the composition of the volatile profiles of natural and washed coffee. Methylpyrazine; 2,3-pentanedione (and its linear and cyclic methylate and dimethyl derivates); furfuryl formate; 5-methyl-2-furfuraldehyde; 2 formyl-1-methyl pyrrole; linalool; maltol; and other compounds ascribable to the classes of furans and pyrazine were identified as being significantly different in the two sets of samples.

**Figure 8:**
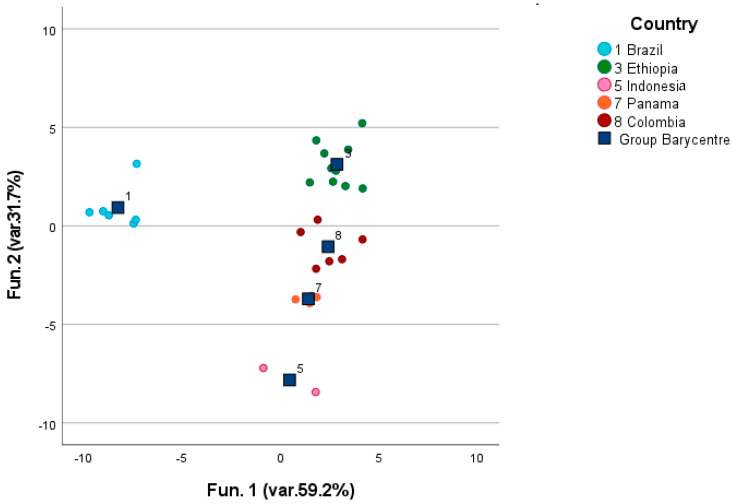
Discrimination of the countries of origin in the dark-roasted coffee subcluster.

In contrast, the countries of origin were best identified when considering only the dark-roasted samples, potentially due to the intense thermal treatments that push the reaction involving fixed compounds in the beans—the source of coffee’s complex aroma profile, the reduction in the number variables, and the variability provided by the post-harvesting processes, may allow features of origins to show their effect in terms of the volatile profile (Figure 8). In this case, 100.0% of the samples were consistently classified and 85.7% of them were confirmed via leave-one-out validation. That is a satisfying result when compared with the outcomes obtained from green coffee (<15%). These outcomes confirm the prevalence of roasting already stated by Zakidou et al. [11] and are also in line with those reported by Caporaso et al. [18], even if, in the present work, the number of samples was increased (n = 30).

The discriminant molecules were 4,6-dimethylpyrimidine; 5-methyl-2-furfuraldhyde; 1-methyl-2-formylpyrrole; 2-furfurylpropan-1-one; benzene-acetaldehyde; 2,5-dimethyl-4-hydroxyfuran-1-one; and 2-methoxy-vinylphenol.

Lastly, in the results obtained, for both country and post-harvesting processes, discriminations are in line with those already assessed for green coffee, confirming that the volatile profile is more suitable for origin rather than post-harvesting processes discriminant models. However, it is useful to identify discriminant compound markers of natural and washed processed coffee in both green and roasted samples, as observed in the *t*-test results.

## 5. Conclusions

To conclude, this work provided a deep overview of the volatile composition of Specialty Arabica coffee, both as green and roasted coffee.

The findings support the generally accepted peculiarities of Specialty coffees that link the aromatic composition to the origin, the post-harvesting process, and the roasting level.

Even if the roasting profiles have a strong impact on the volatile composition of coffee, features deriving from the origin and post-harvesting processes are preserved in roasted coffee because they act as precursors in specific biochemical pathways, potentially helping discriminate among them via the headspace analysis.

It is confirmed that the origin has a stronger impact on the volatile profile than the post-harvesting process. Even when coffee of a single origin is kept for analysis, it is possible to identify the post-harvesting processes.

Further studies are needed to identify the correlation between the volatile profiles of green and roasted coffee and to potentially predict the effect, in terms of the aroma composition, of roasting on a defined green coffee lot.

## Figures and Tables

**Table 1:** List of compounds and related Linear Retention Index (LIR) detected in the green coffee headspace, listed by chemical class.

Alcohols	LRI	Linear Alkanes	LRI
Ethanol	565	Trimethyl-octane	904
3-Methylbutanol	742	n-Decan	1000
2,3-Butanediol	804	2,6-Dimethyloctane	1016
2-Heptanol	873	Tetramethyl-octane	1068
Octanol	1007	3-Methylnonane	1064
Phenylethyl alcohol	1126	Tetramethyl-heptane	1026
**Acids**	**LRI**	Undecane	1100
Acetic acid	595	3-Methylundecane	1176
3-Metyl butanoic acid (isovaleric acid)	856	Dodecane	1200
2-Methyl butanoic acid	863	3-Methyl-tridecane	1374
3-Methyl-but-2-en-oic acid	884	Tetradecane	1400
Pentanoic acid (valeric acid)	894	Pentadecane	1500
3-Methyl-pentanoic acid	903	**Terpenes**	**LRI**
**Esters**	**LRI**	β-Pinene	898
Methyl ester isovaleric acid	809	Farnesane (2,6,10-trimethyl-dodecane)	1241
2-Methyl-ethyl ester butanoic acid	840	α-Pinene	930
3-Methyl-ethylestere butanoic acid (ethyl ester isovaleric acid)	843	Carene	1010
Propanoic acid methyl hexyl ester	1058	D-limonene	1023
**Aldehydes**	**LRI**	α-Linalool	1104
Hexanal	798	**Pyrazines e pyridines**	**LRI**
Benzaldehyde	907	Methylpyrazine	832
Nonanal	1110	3-Methoxypyrazine-2-isobutyle	1181
**Ketones**	**LRI**	**Furans**	**LRI**
1-Methoxy-2-propanone	582	2-Pentylfuran	985
Cyclopentanone-2-sec-butyl	1221	Furfuryl-alcohol	831
6,10,14-Trimethyl-2-pentadecanone	1752	**Contaminants**	**LRI**
Diphenyl-propane	1654	Ethylbenzene	848
**Alkaloids**	**LRI**	Tributyl-phosphate	1602
Caffeine	1772	Phthalic acid dibutyl-ester	1764
		Diethyl phthalate	1558

**Table 2:** List of compounds and related Linear Retention Index (LIR) detected in the roasted coffee headspace, listed by chemical class.

Furans and Derivatives	LRI	Pyrazole	LRI
Dihydro 2-methyl-3-furanone	820	Pyrazole-3,4-pyrimidine	1227
Furfural	842	**Pyridines**	**LRI**
Furfuryl alcohol	831	Pyridine	776
Furfuryl formate	890	1-Acetyl-1,4-dihydropyridine	990
3-Furanone-2,5-dimethyl-2-((hydroxy-1-acetyl) ethyl)	930	**Pyrazines**	**LRI**
5-Methylfurfural	942	Methylpyrazine	832
Furfuryl acetate	955	4,6-Dimethylpyrimidine	906
1-Propanone-2-furanyl	968	2-Ethyl-6-methylpyrazine	959
2,2-Bifuran	1023	2-Ethyl-5-methylpyrazine	962
Alanine N ethyl furfuryl ester	1072	2-Methyl-6-vinyl pyrazine	982
Furan, 2,2′-methylenebis	1117	2-Acetyl pyrazine	998
4-Hydroxy-2,5-dimethyl-3(2H)-furanone	1128	3-Ethyl-2,5-dimethyl pyrazine	1112
5-Methyl-2,2-dimethylene furan	1217	1-(6-Methyl-2-pyrazinyl) ethenone	1163
Furfuryl methyl disulfide	1258	2-Acetyl-3-methyl pyrazine	1155
Furfuryl-3-methylbutanoate	1262	5-Methyl-6,7-dihydro-5H-cyclopenta pyrazine	1181
3-Phenylfuran	1272	3,5-Diethyl-2-methylpyrazine	1197
6-(5-Methyl-furan-2-yl)-hexan-2-one	1291	2-Methyl-5(1-propenyl) pyrazine	1242
2,2′-Difurylmethane	1130	3,5-Dimethyl-2-acetyl pyrazine	1265
Furfuryl methylamine	1401	6-Methyl-2-isoamyl pyrazine	1294
**Ketones**	**LRI**	**Acids**	**LRI**
2,3-Pentanedione	590	Isovaleric acid	856
3-Methyl-1,2-cyclopentanedione	1013	2-Methylene-4-hydroxy butyric acid	1028
2,4-Dimethyl-1,3-cyclopentanedione	1050	**Pyrroles**	**LRI**
Methyl acetyl acetone	1143	2-Formyl-1-methylpyrrole	965
3-Ethyl-2-hydroxy-cyclopentene-1-one	1135	Pyrrole-2-carboxyaldehyde	1007
**Terpenes**	**LRI**	Pyrrole-2-acetonitrile-1-methyl	1150
Linalool	1104	**Alcohols**	**LRI**
Damascenone (2,6,6-trimethylcyclohexa-1,3-diene)	1379	Maltol	1157
4-Ethyl-2-methoxy phenol	1320		
2-Methoxy-4-vinyl phenol	1353		

## Data Availability

Research data are available in the article’s supplementary materials and on request from the authors.
